# Acceptability of Pre-Exposure Prophylaxis among Men Who Have Sex with Men and Transgender Women in Northern Thailand

**DOI:** 10.1371/journal.pone.0076650

**Published:** 2013-10-08

**Authors:** Daniel Yang, Chonlisa Chariyalertsak, Antika Wongthanee, Surinda Kawichai, Kriengkrai Yotruean, Pongpun Saokhieo, Thomas Guadamuz, Voravit Suwanvanichkij, Chris Beyrer, Suwat Chariyalertsak

**Affiliations:** 1 University of California San Francisco, San Francisco, California, United States of America; 2 Johns Hopkins Bloomberg School of Public Health, Baltimore, Maryland, United States of America; 3 Research Institute for Health Sciences, Chiang Mai University, Chiang Mai, Thailand; 4 Chiang Mai Provincial Public Health Office, Thailand Ministry of Public Health, Chiang Mai, Thailand; 5 Mahidol University, Center for Health Policy Studies, Faculty of Social Sciences and Humanities, Nakhon Pathom, Thailand; 6 University of Pittsburgh Graduate School of Public Health, Pittsburgh, Pennsylvania, United States of America; 7 Faculty of Medicine, Chiang Mai University, Chiang Mai, Thailand; University of Washington, United States of America

## Abstract

**Background:**

Northern Thailand has a high burden HIV epidemic among MSM and TG. Oral pre-exposure prophylaxis (PrEP) with tenofovir-emtricitabine has demonstrated efficacy in preventing HIV among MSM and TG in Chiang Mai, Thailand. Determinants of PrEP acceptability are needed to gauge the potential uptake of this prevention strategy.

**Methods:**

From January to February 2012, 238 MSM and TG participants, who self-reported as HIV-uninfected or of unknown status, completed a self-administered survey on hand-held computers. Participants were recruited by venue-day-time sampling and asked to rate their likelihood of using oral PrEP for HIV prevention with an efficacy of 50%. PrEP acceptability was defined as being “very likely” to use PrEP. Odds ratios and 95% CIs were calculated to identify correlates of acceptability.

**Results:**

131 MSM and 107 TG responded, with mean ages of 23.7 and 21.8, respectively. 24% of MSM engaged primarily in receptive anal sex vs. 74% of TG. 21% of MSM and 44% of TG reported regular medication use. Prior awareness of PrEP was high at 66% among both MSM and TG respondents. 41% of MSM and 37% of TG were "very likely" to use PrEP. Among MSM, factors associated with PrEP acceptability included a prior history of STIs (AOR 4.6; 95%CIs 1.7-12.6), previous HIV testing (AOR 2.4 95%CIs 1.1-5.3), regularly planned sex (AOR 2.8 95%CIs 1.1-7.2), and infrequent sex (AOR 2.9 95%CIs 1.3-6.3). Among TG, factors associated with acceptability included prior awareness of PrEP (AOR 3.3; 95%CIs 1.2-9.0) and having private insurance (AOR 5.0; 95%CIs 1.3-19.0).

**Conclusion:**

MSM and TG in Northern Thailand are distinct groups in terms of sexual behaviors, patterns of medication use, and correlates of PrEP acceptability. Efforts to maximize PrEP uptake should include expanded HIV testing services and the provision of financial subsidies to reduce the cost of PrEP.

## Introduction

Northern Thailand is experiencing a severe epidemic of HIV among men who have sex with men (MSM) and transgender women (TG), persons born biologically male and expressing female gender identity. A 2009 analysis in Chiang Mai, Thailand, estimated an HIV prevalence rate of 17% among gay men and 9% among TG [1], despite an estimated prevalence of 1.3% in the general adult Thai population [2]. A recent report among MSM in Bangkok found an HIV incidence rate of 5.9/100 person-years among a cohort of 1,744 MSM followed from 2006-2011 [3].

To counter the high rates of HIV infection, new prevention strategies are arguably needed. One strategy that has demonstrated efficacy in reducing incident HIV infections in MSM and TG is oral pre-exposure prophylaxis (PrEP) with tenofovir-emtricitabine (TDF-FTC). The Pre-Exposure Prophylaxis (iPrEx) trial, involving nearly 2,500 HIV-uninfected MSM and TG at high risk of acquiring HIV, demonstrated that daily use of TDF-FTC reduced HIV infections by 44% versus placebo [4]. Subsequent analyses have also demonstrated the efficacy of oral PrEP in preventing HIV infection in high-risk heterosexual men and women [5,6] and, in July 2012, the US Food and Drug Administration (FDA) approved the use of daily, oral TDF-FTC as PrEP for high-risk HIV-negative sexually active adults. The Centers for Disease Control and Prevention (CDC) have also published interim guidance for clinicians on the use of PrEP for HIV prevention in MSM and heterosexual adults

 [7,8].

Although demonstrated to be efficacious, the effectiveness of PrEP is dependent on uptake, acceptability, and adherence in those at risk. Published data suggests that awareness of PrEP remains generally low [9-17], and that many populations at risk would be willing to take PrEP if it was effective and accessible [9-11,16-21]. In surveys of MSM both in the US and low-income countries, PrEP was found to be more acceptable among those reporting high-risk behaviors such as unprotected anal intercourse [11,16,22], inconsistent condom use [16], recent recreational drug use [10], and a history of sexually transmitted infections [23]. Increasing PrEP acceptability may also correlate in those with lower-income [14], lower educational status [9,11], and younger age [11,14,24]. Significant barriers to PrEP acceptability include the cost of medication [19,21-23], fear of risk compensation or sexual dis-inhibition [19,20,22], the burden of taking a daily medication [19], and concerns over side effects [19,21,22].

To date, the vast majority of published data on PrEP acceptability has come from studies of MSM. There have been no systematic evaluations exploring the attitudes of TG towards PrEP. A sub-group analysis of the iPrEX trial data demonstrated no efficacy of oral PrEP among those who self-identified as "trans" or who reported using female sex hormones, with the same number of incident HIV infections (11) in both the FTC-TDF and placebo groups [25]. The difference in PrEP efficacy between MSM and TG populations is not well understood. It is conjectured to be the result of differences in patterns of PrEP use or sexual behaviors, a potential effect of female sex hormones on drug transport in the mucosa, or a consequence of chance [25].

Epidemiological studies have demonstrated that the TG community in Thailand is a distinct population from MSM, both with regard to sexual behaviors and medication use. In one analysis, 97% of Thai TG reported strictly receptive anal sex compared to 34% of self-identified gay men [1]. Thai TGs were also much more likely to use female hormones; in another analysis nearly 90% of TG reported a lifetime history of hormone use, and over 50% reported daily use [26]. Given the dearth of reliable data on this population, particularly with regard to attitudes towards PrEP, this study was designed to explore these issues in the TG population of Chiang Mai, Northern Thailand.

The objectives of this current study are to investigate the prevalence of PrEP awareness post-iPrEX, patterns of medication use, and PrEP acceptability among MSM and TG populations in Northern Thailand, utilizing a sub-analysis to compare the two populations. Determining demographic and behavioral correlates of PrEP acceptability in each population will be useful for identifying potential users most likely to benefit from PrEP and for developing effective strategies for PrEP roll-out in Thailand.

## Methods

### Ethics Statement

All participants provided verbal informed consent. Verbal informed consent was obtained in order to ensure the anonymity of participants, eliminating the risk that signatures could be linked to responses. Consent was documented electronically by participants checking a consent box on a hand-held computer. All study procedures were reviewed and approved by the Chiang Mai Provincial Public Health Office Institutional Review Board.

### Participants and Procedures

The current survey was adapted from a previous tool used to assess HIV incidence and risk factors among MSM and TG presenting for HIV testing and pre-screening for the iPrEX trial at the PIMAN clinic in Chiang Mai, Thailand [1]. Between January and February 2012, 238 participants completed a self-administered survey on handheld computers. Eligible participants were biologic males at birth, at least 18 years of age, HIV-uninfected or of unknown status on self-report, and had anal or oral sex with a man in the preceding 6 months.

### Recruitment

Participants were recruited using venue-day-time sampling, previously used by the Chiang Mai Provincial Public Health Office for HIV surveillance among local MSM and TG communities [26,27]. Methods have been described in detail elsewhere [28-31]. Survey venues in Chiang Mai were randomly selected from a universe of potential venues identified by the Public Health Office. The venues included popular entertainment areas such as bars, discos, saunas, and massage parlors, as well as educational institutions such as universities and vocational schools. Specific days and times when MSM and TG were known to congregate at each selected venue were randomly chosen. Field staff visited the venues at the specified time period and systematically approached individuals for participation in the study. Potential participants, after providing verbal informed consent, were asked to complete a brief screening interview on handheld computers. Eligible participants went on to complete the anonymous 20-minute survey.

### Measures

The survey collected participant data on demographic characteristics, substance and female hormone use, sexual behaviors, STI history, and previous history of HIV testing. Additionally, the current survey collected data on medication management skills and PrEP awareness and acceptability. PrEP was described to participants in the survey, defined as “the regular use of HIV medicines, called antiretrovirals, by HIV-negative individuals to reduce their risk of infection with HIV.”

### Primary Outcome

The primary outcome of this study was PrEP acceptability, assessed by a 4-point likert scale. Participants were asked, “If PrEP was 50% effective in preventing HIV infection, how likely are you to use PrEP as an HIV prevention method?” Participants responded as “very likely,” “somewhat likely,” somewhat unlikely,” or “very unlikely” to use PrEP. PrEP efficacy was defined as 50%, to approximate overall efficacy according to the results of the iPrEX trial [4]. Responses were then dichotomized, with PrEP acceptability conservatively defined as being "very likely" to use the intervention.

### Data Analysis

All data was analyzed using STATA version 10.0 (STATA Corp, College Station, TX). Descriptive statistics were used to examine sample characteristics and patterns of PrEP awareness and acceptability. Pearson Chi-Square tests were conducted to identify significant associations with PrEP acceptability. Crude odds ratios and 95% confidence intervals were calculated. Variables with a p-value <0.10 were retained for multivariate logistic regression analysis. Using a backwards stepwise procedure, non-significant variables (p>0.05) were removed from the final multivariate model. Crude and adjusted odds ratios and 95% confidence intervals were then calculated.

## Results

### Demographics

In all, 326 individuals completed the screening questionnaire at 17 venues around Chiang Mai City. There were 238 individuals (73%), 131 MSM and 107 TG, who met eligibility criteria and completed the survey. The demographic characteristics of MSM and TG participants are summarized in Table 1. Most MSM participants were recruited from entertainment venues (66%), whereas most TG participants were recruited from educational venues (67%). MSM ages ranged from 18 to 49 years with mean age of 23.7. TG ages ranged from 18 to 33 years with mean age of 21.8. Thirty-eight percent of MSM and 62% of TG were enrolled in or had completed a bachelor’s degree or higher. Eighty-four percent of MSM respondents were of Thai ethnicity; the remainder were of other ethnic backgrounds, particularly Shan, Burman, and Akha. All TG respondents were Thai. Two percent of the respondents needed assistance from interviewers to complete the questionnaire due to an inability to read Thai or use the hand held computer.

**Table 1:** Demographic Characteristics of MSM and TG in Chiang Mai, 2012.

	MSM (n=131)		TG (n=107)	
Characteristics and Risk Behaviors	**N**	**%**	**N**	**%**
**Venue Recruitment**				
Educational Institution	32	*24*	72	*67*
Public Place	12	*9*	23	*22*
Entertainment Venue	87	*66*	12	*11*
*χ^2^(2)=73.99, p<0.001*				
**Age (years); Mean (Range)**	*23.7 (18-49)*		*21.8 (18-33)*	
<25	85	*65*	89	*83*
≥25	46	*35*	18	*17*
*χ^2^(1)=10.02, p=0.002*				
**Ethnicity**				
Thai	110	*84*	107	*100*
Non-Thai	21	*16*	0	*0*
*χ^2^(1)=18.81, p<0.001*				
**Education Level**				
Secondary or Less	34	*26*	5	*5*
Vocational School	47	*36*	36	*34*
Bachelors or Higher	50	*38*	66	*62*
*χ^2^(2)=23.04, p<0.001*				
**Occupation**				
Student/Unemployed	59	*45*	86	*80*
Employed	72	*55*	21	*20*
*χ^2^(1)=30.89, p<0.001*				
**Monthly Income**				
<5,000 baht (~167 USD)	39	*30*	53	*50*
≥5,000 baht	92	*71*	54	*51*
*χ^2^(1)=9.7, p=0.002*				
**Health Insurance**				
30 baht health insurance†	74	*56*	73	*73*
Social Security	44	*34*	39	*36*
Private Insurance	26	*20*	13	*12*
Other‡	50	*39*	4	*4*
No Health Insurance	10	*8*	3	*3*
*χ^2^(4)=33.72, p<0.001*				
**Sexual Orientation** (**MSM**)*				
Heterosexual, Straight	17	*13*	NA	
Bisexual	21	*16*	NA	
Homosexual, Gay	93	*71*	NA	
**Surgery** (**TG**)§				
Sex Reassignment Surgery	NA		6	*6*
Orchiectomy	NA		5	*5*
Neither Sex Reassignment nor Orchiectomy	NA		96	*90*

† 30 baht health insurance is a universal coverage scheme introduced by the Thai government in 2001, with a standard co-payment of 30-baht (~1 USD)

‡ Other health insurance types include welfare for government officers and health insurance for foreigners.

* Options listed for sexual orientation include heterosexual, bisexual, gay, or TG.

§ Only TG respondents were asked about their surgical history

### Sexual Orientation, Gender Identity, Behavior, and Substance Use

Among MSM surveyed, 13% self-identified as heterosexual, 16% as bisexual, and 71% as gay. Among those who self-identified as TG, 6% had undergone sex reassignment surgery, 5% had undergone orchiectomy alone, and around 90% had undergone neither sex reassignment surgery nor orchiectomy (Table 1). The sexual behaviors and substance use patterns of MSM and TG participants are described in Table 2. Seventy-four percent of TG primarily engaged in receptive anal sex, compared to 24% of MSM. Eight percent of both MSM and TG participants reported a lifetime history of substance use. The mean number of *regular* sex partners in the preceding 6 months was 1.5 for MSM and 1.8 for TG. The mean number of *casual* sex partners in the preceding 6 months was 3.6 for MSM and 1.8 for TG. Sixteen percent of MSM and 19% of TG reported unprotected anal sex with a casual male partner in the preceding 6 months.

**Table 2:** Sexual Behaviors, STIs, and Substance Use among MSM and TG in Chiang Mai, 2012.

**Usual Anal Sex Position**				
Insertive	42	*32*	6	*6*
Receptive	32	*24*	79	*74*
Versatile	57	*44*	22	*21*
*χ^2^(2)=60.60, p<0.001*				
**Number of Regular Sex Partners, (last 6 m); Mean (Range)**	*1.5 (0-18)*		*1.8 (0-30)*	
0	38	*29*	27	*25*
1	45	*34*	49	*46*
≥2	48	*37*	31	*29*
*χ^2^(2)=3.30, p=0.19 (NS)*				
**Condom Use with Male Regular Sex Partners** ¶				
Always	32	*24*	34	*32*
Not Always	32	*24*	37	*35*
*χ^2^(1)=0.06, p=0.81 (NS)*				
**Number of Casual Sex Partners (last 6 m); Mean (Range)**	*3.6 (0-81)*		*1.8 (0-20)*	
0	42	*32*	41	*38*
1	23	*18*	29	*27*
≥2	66	*50*	37	*35*
*χ^2^(2)=6.52, p=0.038*				
**Condom Use with Male Casual Sex Partners**°				
Always	51	*39*	38	*36*
Not Always	21	*16*	20	*19*
*χ^2^(1)=0.92, p=0.52 (NS)*				
**Received Money, Gifts, Valuables for Sex (ever)**				
Yes	40	*31*	18	*17*
No	91	*70*	89	*83*
*χ^2^(1)=6.01, p=0.014*				
**Planned Sex**				
Yes	29	*22*	28	*26*
No	102	*78*	79	*74*
*χ^2^(1)=0.53, p=0.47 (NS)*				
**Frequency of Sex**				
>1 time per month	72	*55*	59	*56*
≤1 time per month	59	*45*	48	*45*
*χ^2^(1)=0.00, p=0.98 (NS)*				
**Drug Use (Ever)**				
Yes	10	*8*	9	*8*
No	121	*92*	98	*92*
*χ^2^(1)=0.05, p=0.83 (NS)*				
**Sex Under the Influence of Drugs (ever)**				
Yes	16	*12*	13	*12*
No	115	*88*	94	*88*
*χ^2^(1)=0.00, p=0.99 (NS)*				
**Previous HIV testing** (**ever**)				
Yes	75	*57*	53	*50*
No	56	*43*	54	*51*
*χ^2^(1)=1.41, p=0.24 (NS)*				
**Previous STI (ever)♦**				
Yes	27	*21*	8	*8*
No	104	*79*	99	*93*
*χ^2^(1)=8.10, p=0.004*				

¶ Only participants that reported having anal sex with at least 1 male regular partner were asked about condom use.

° Only participants that reported having anal sex with at least 1 male casual partner were asked about condom use.

♦ Self-reported, symptomatic STIs – participants were asked if they have ever experienced unusual penile fluid, irritative urinary symptoms, ulcers, warts or penile rashes.

### Medication Management Skills

Table 3 describes the medication management skills of MSM and TG in Chiang Mai. Twenty-one percent of MSM and 44% of TG reported regular oral medication use (p<0.001). Six percent of MSM and 45% of TG reported current female hormone use (p<0.001). Twenty-one percent of MSM and 19% of TG responded that they were "very confident" in their ability to take an oral medicine daily for one year. Of those that reported regular oral medication use, 68% of MSM (19/28) and 73% of TG (35/48) feared an interaction between PrEP and their other medications (data not shown).

**Table 3:** Medication Management Skills and PrEP Awareness and Acceptability among MSM and TG in Chiang Mai, 2012.

**Regular Medicine Use**				
Yes	28	*21*	48	*45*
No	103	*79*	59	*55*
*χ^2^(1)=14.95, p<0.001*				
**Female Hormone Use (Ever)**				
Yes	28	*21*	72	*67*
No	103	*79*	35	*33*
*χ^2^(1)=50.97, p<0.001*				
**Current Hormone Use**				
Yes	8	*6*	48	*45*
No	20	*15*	24	*23*
*χ^2^(1)=11.87, p<0.001*				
**Confidence to take oral medicines daily for 1 year**				
Very confident	28	*21*	20	*19*
Not very confident	103	*79*	87	*81*
*χ^2^(2)=0.17 p=0.68 (NS)*				
**Prior PrEP Awareness**				
Yes	86	*66*	71	*66*
No	45	*34*	36	*34*
*χ^2^(1)=0.01, p=0.91 (NS)*				
**PrEP Acceptability**				
Yes	54	*41*	40	*37*
No	77	*59*	67	*63*
*χ^2^(1)=0.36, p=0.55 (NS)*				

### PrEP Knowledge and Acceptability

Sixty-six percent of both MSM and TG respondents (p=0.91) had prior knowledge or PrEP (Table 3). Participants heard of PrEP primarily from friends (53%) and/or health care providers (54%, data not shown). PrEP acceptability was 41% among MSM and 37% among TG (p=0.55, Table 3).

Table 4 shows the results of bivariate and multivariate analysis of variables associated with PrEP acceptability. In bivariate analysis of MSM participants, PrEP acceptability was associated with having zero regular partners in the preceding 6 months (OR 2.25, P=0.04) vs. one or more partners, regularly planned sex (OR 2.83, P=0.01) vs. unplanned sex, infrequent sex (once per month or less, OR 2.36, P=0.02) vs. two or more sexual encounters per month, a lifetime history of STIs (OR 3.78, P<0.01) vs. no history of STIs, a lifetime history of HIV testing (OR 1.95, P=0.07) vs. no history of HIV testing, age 25 years or older (OR 2.30, P=0.02) vs. age less than 25 years, and being “very confident” in the ability to take daily, oral medicines for 1 year (OR 2.63, P=0.01) vs. not being “very confident”. In contrast, receptive anal sex positioning (OR 0.47, P=0.08) was negatively associated with PrEP acceptability vs. insertive or versatile positioning.

**Table 4:** Bivariate and Multivariate Analysis: Correlates of PrEP Acceptability among MSM and TG in Chiang Mai, 2012.

	MSM (n=131)				TG (n=107)			
Variables	N	Accept PrEP (%)	Crude OR(95% CI)	aOR (95% CI)	N	Accept PrEP (%)	Crude OR (95% CI)	aOR (95% CI)
**Age (years)**								
<25	85	34%	1.00		89	38%	1.00	
≥25	46	54%	2.30 (1.10a-4.79)† *	‡	18	33%	0.81 (0.28-2.36)	
**Private Insurance**								
Yes	26	50%	1.56 (0.6-4.05)		13	62%	**3.10 (0.81-12.94**)**†**	**5.00 (1.32-19.01**)*
No	105	39%	1.00		94	34%	1.00	1.00
**Usual Anal Sex Position**								
Receptive	32	28%	0.47 (0.17-1.19)†	‡	79	39%	0.44 (0.51-3.87)	
Insertive/Both	99	45%	1.00		28	32%	1.00	
**Number of Regular Partners (last 6 m)**								
0	38	55%	1.00	‡	27	33%	1.00	
1	45	33%	0.40 (0.15-1.08)† *		49	39%	1.27 (0.43-3.89)	
≥2	48	38%	0.49 (0.19-1.26)†		31	39%	1.26 (0.38-4.28)	
**Alcohol Use (last 6 m)**								
Yes	113	41%	0.86 (0.28-2.71)		84	42%	2.57 (0.81-9.64)†	‡
No	18	44%	1.00		23	22%	1.00	
**Planned Sex**								
Yes	29	62%	**2.83 (1.12-7.12**)†*	**2.84 (1.13-7.16**)*	28	39%	1.11 (0.41-2.94)	
No	102	35%	1.00	1.00	79	37%	1.00	
**Frequency of Sex**								
>1 time per month	72	32%	1.00	1.00	59	39%	1.00	
≤1 time per month	59	53%	**2.36 (1.09-5.11**)†*	**2.85 (1.29-6.28**)*	48	35%	0.86 (0.36-2.03)	
**Drug use before Sex with Male or TG Partner (ever)**								
Yes	16	50%	1.50 (0.45-4.93)		13	8%	0.12 (0.003-0.87)† *	‡
No	115	40%	1.00		94	41%	1.00	
**STI History (ever)**								
Yes	27	67%	**3.78 (1.42-10.47**)†*	**4.63 (1.70-12.60**)*	8	25%	0.54 (0.05-3.21)	
No	104	35%	1.00	1.00	99	38%	1.00	
**HIV Testing (ever)**								
Yes	75	48%	**1.95 (0.89-4.29**)**†**	**2.39 (1.07-5.32**)*	53	40%	1.21 (0.51-2.85)	
No	56	32%	1.00	1.00	54	35%	1.00	
**Confidence to take oral medicines daily for 1 year**								
Very confident	36	58%	2.63 (1.12-6.24)† *	‡	20	50%	1.9 (0.63-5.70)	
Not very confident	95	35%	1.00		87	34%	1.00	
**Prior PrEP Awareness**								
Yes	86	44%	1.43 (0.64-3.26)		71	44%	**2.33 (0.89-6.42**)**†**	**3.30 (1.21-8.95**)*
No	45	36%	1.00		36	25%	1.00	1.00

† P < 0.10 in bivariate χ^2^ tests and qualified to be entered into the multiple logistic regression model

* P < 0.05

‡ Variable excluded from final multivariate model in the backward stepwise selection procedure, adjusted P > 0.05

In the final multivariate logistic regression model of MSM participants, PrEP acceptability was associated with having a lifetime history of STIs (aOR 4.63, P<0.01), previous HIV testing (aOR 2.39, P=0.03), regularly planned sex (aOR 2.84, P=0.03), and infrequent sex (once per month or less, aOR 2.85, P<0.01).

For TG participants, on bivariate analysis, alcohol use in the preceding 6 months (OR 2.57, P=0.08) vs. no alcohol use in the last 6 months, having private insurance (OR=3.10, P=0.06) vs. not having private insurance, and prior knowledge of PrEP (OR 2.33, P=0.06) vs. no prior knowledge of PrEP were positively correlated with PrEP acceptability, whereas a lifetime history of sex with a male or TG partner under the influence of drugs was negatively associated with PrEP acceptability (OR 0.12, P=0.02) vs. never having had sex with a male or TG partner under the influence of drugs.

In the final multivariate logistic regression model of TG participants, having private insurance (aOR 5.00, P=0.02) and prior knowledge of PrEP (aOR 3.30, P=0.02) were independently correlated with PrEP acceptability.

## Discussion

To our knowledge, this is the first study to explore PrEP awareness and acceptability among MSM and TG populations in Thailand. Awareness of PrEP was generally high (66%) in both MSM and TG participants, and acceptable for 41% of MSM and 37% of TG participants. Independent correlates of PrEP acceptability among MSM included having had previous HIV testing, a lifetime history of STIs, infrequent sex, and regularly planned sex. Independent correlates among TG included prior awareness of PrEP and having private insurance.

The relatively high prior awareness of PrEP for both MSM and TG participants is consistent with a study of MSM couples in San Francisco, which was also conducted after the publication of the iPrEX trial [22]. . However, qualitative data from Saberi’s study suggests that the high prevalence of PrEP awareness is likely an overestimate since more than one quarter of participants had mistaken PrEP with post-exposure prophylaxis (PEP). It is possible that a similar confusion in our study may account for an overestimate of PrEP awareness.

Acceptability of PrEP, defined as individuals who reported being “very likely” to use PrEP, was similar between MSM and TG groups (around 40%), a figure consistent with several previously reported studies [22,23]. However, other published studies have reported higher rates of PrEP acceptability, ranging from 44%-86% [9-11,14,16-18]. This discrepancy may be a consequence of how PrEP efficacy is defined. Reports have demonstrated lower PrEP acceptability in the setting of lower perceived efficacy [19,20]. Most previous surveys did not report a hypothetical PrEP efficacy; however, those that did, described PrEP as being 75-95% effective in preventing HIV infections [10,20] compared to our stated efficacy of 50%. Consequently, the results of this study may reflect a more realistic level of PrEP acceptability given the current evidence of partial efficacy.

Correlates for PrEP acceptability among MSM include previous HIV testing and a lifetime history of STIs. These findings are consistent with previous studies which also identified HIV testing [24] and a history of STIs [23] as independent correlates of PrEP acceptability. These two correlates may reflect increased knowledge of HIV and a greater concern for one’s risk of contracting HIV or other STIs. Furthermore, a history of HIV testing and STIs may also reflect greater access to and familiarity with healthcare services and may be a promising sign for PrEP roll-out since regular HIV testing and follow-up visits will be an essential component of PrEP implementation [8]. Given the correlation between HIV testing and PrEP acceptability, an expansion of voluntary HIV counseling and testing services for at-risk MSM may improve PrEP uptake and reduce incident HIV infections in this population.

Infrequent sex and planned sex, which represent lower sexual risk behaviors, were positively associated with PrEP acceptability among MSM. This is in contrast to previously published studies that have found PrEP acceptability to be correlated with higher risk behaviors [11,16-18]. The correlation of PrEP acceptability with infrequent and planned sex may have implications for the dosing regimen of PrEP. Intermittent PrEP, administered before and after sex, may be better suited for MSM who have infrequent and planned sex than daily PrEP. This dosing strategy may also result in improved adherence, lower costs, decreased pill burden, and a smaller risk of side effects [32]. A clinical trial in South Africa and Thailand is currently underway to evaluate adherence to and acceptability of fixed-interval and coitally-dependent intermittent PrEP [33].

In contrast to MSM, correlates of PrEP acceptability among TG were prior PrEP awareness and having private insurance. This suggests that efforts to increase awareness of PrEP may improve uptake for TG populations. However, with PrEP awareness being relatively high among this population (66%), educational campaigns may also play an important role in improving the accuracy of PrEP understanding and minimizing confusion of PrEP with PEP and other biomedical HIV prevention and treatment modalities. Another important issue to consider is potential drug interactions between PrEP and other medications. In our study, we found that nearly half of TG participants took oral medicines regularly and nearly three-fourths of these participants feared a drug interaction between PrEP and other medicines, particularly female hormones. As a result, it will be important to clearly address the issue of treatment interactions in educational campaigns and leverage the high prevalence of regular medication use into improved adherence for PrEP.

Previous studies have identified out-of-pocket-cost as a major barrier to PrEP acceptability [19-23]. The correlation between private insurance and PrEP acceptability in TG likely reflects concern in this population about the cost of PrEP, which is not currently available through any of Thailand’s public health programs.

However, these findings are preliminary and there are several limitations in this analysis. Although the use of venue-day-time sampling provides a systematic sample of a hard-to-reach population, this recruitment method is still subject to sampling bias. Venue-day-time-sampling recruits participants found in venues during high traffic periods and thus the findings of this analysis may not reflect the experiences of all MSM and TG in Chiang Mai, particularly those who do not attend venues. As many of the sampled venues are locations where MSM and TG attend to seek sexual partners, the prevalence of HIV risk behaviors may be overestimated compared to the general population of MSM and TG. Our findings may also be subject to social desirability bias, which may have led to an overestimate of PrEP acceptability despite the use of hand-held computers to maximize privacy and anonymity. This survey also measured intent-to-use PrEP and thus may not reflect actual behaviors once PrEP becomes more widely available, especially if PrEP is combined with other interventions such as regular follow-up appointments and quarterly HIV testing [8,34].

Yet these limitations should not detract from one significant finding of this analysis, that TG populations are a different risk population than MSM, particularly with regard to sexual behaviors, patterns of medication use, and correlates of PrEP acceptability. TG may have higher rates of receptive anal sex but otherwise reported lower sexual risk behaviors compared to MSM, and were more likely to use other medications, particularly female hormones. This may have implications on PrEP expansion in this population, particularly given the widespread concern over medication interactions among TG and findings of the iPrEx trial, where the efficacy of PrEP was markedly lower in TG compared to other MSM [25]. Additional research is urgently warranted to further explore these issues.

This study sought to measure the prevalence of PrEP awareness and acceptability post-iPrEX and determine the behavioral and demographic correlates associated with increased PrEP acceptability among MSM and TG populations in Northern Thailand. Findings from this study suggest that maximizing future PrEP uptake among MSM and TG populations in Thailand may require targeted educational campaigns to improve awareness and accuracy of PrEP understanding, in addition to further access to HIV voluntary counseling and testing services for at risk MSM and TG populations, particularly given the severe HIV epidemic in Thailand in both of these populations. As a resource-limited setting, reduction of out-of-pocket costs for PrEP would also be an important consideration in Thailand for MSM and particularly TG, who often use other medications.
